# Comprehensive analysis of senescence-associated genes in sepsis based on bulk and single-cell sequencing data

**DOI:** 10.3389/fmolb.2023.1322221

**Published:** 2024-01-08

**Authors:** Linfeng Tao, Yue Zhu, Lifang Wu, Jun Liu

**Affiliations:** ^1^ Gusu School of Nanjing Medical University, Department of Critical Care Medicine, The Affiliated Suzhou Hospital of Nanjing Medical University, Suzhou Municipal Hospital, Suzhou Clinical Medical Center of Critical Care Medicine, Suzhou, China; ^2^ Department of Breast and Thyroid Surgery, The Affiliated Suzhou Hospital of Nanjing Medical University, Gusu School, Nanjing Medical University, Suzhou, China; ^3^ Department of Critical Care Medicine of Kunshan Third People’s Hospital, Suzhou, China

**Keywords:** sepsis, senescence-associated genes, biomarker, WGCNA (weighted gene co-expression network analyses), single-cell analysis

## Abstract

**Background:** Sepsis is a pathological state resulting from dysregulated immune response in host during severe infection, leading to persistent organ dysfunction and ultimately death. Senescence-associated genes (SAGs) have manifested their potential in controlling the proliferation and dissemination of a variety of diseases. Nevertheless, the correlation between sepsis and SAGs remains obscure and requires further investigation.

**Methods:** Two RNA expression datasets (GSE28750 and GSE57065) specifically related to sepsis were employed to filter hub SAGs, based on which a diagnostic model predictive of the incidence of sepsis was developed. The association between the expression of the SAGs identified and immune-related modules was analyzed employing Cell-type Identification By Estimating Relative Subsets Of RNA Transcripts (CIBERSORT) and Microenvironment Cell Populations-counter (MCP-counter) analysis. The identified genes in each cohort were clustered by unsupervised agreement clustering analysis and weighted gene correlation network analysis (WGCNA).

**Results:** A diagnostic model for sepsis established based on hub genes (IGFBP7, GMFG, IL10, IL18, ETS2, HGF, CD55, and MMP9) exhibited a strong clinical reliability (AUC = 0.989). Sepsis patients were randomly assigned and classified by WGCNA into two clusters with distinct immune statuses. Analysis on the single-cell RNA sequencing (scRNA-seq) data revealed high scores of SAGs in the natural killer (NK) cells of the sepsis cohort than the healthy cohort.

**Conclusion:** These findings suggested a close association between SAGs and sepsis alterations. The identified hub genes had potential to serve as a viable diagnostic marker for sepsis.

## Introduction

Sepsis is characterized by impaired immunomodulation that could cause complex and heterogeneous organ dysfunction (Angus and van der Poll, 2013). According to a report in 2017, approximately 48.9 million people were diagnosed with sepsis, resulting in more than 11 million deaths and accounting for 20% of the global death cases (Rudd et al., 2020). Although clinical interventions such as antimicrobial drugs, intravenous fluid administration, and comprehensive organ support have been advanced significantly, sepsis-related mortality continues to increase (Gavelli et al., 2021). Previous research has indicated that biomarkers play a crucial role in the diagnosis of sepsis, early detection of organ failure, risk assessment, prognostic prediction, and development of care plans (Bosmann and Ward, 2013). Hence, discovering sepsis-associated biomarkers is of great importance for a timely detection of sepsis.

Cellular senescence is commonly known as regular cell division in response to various cellular stresses or DNA damage, accompanied by pro-inflammatory responses, dysfunctional mitochondria, and shortened telomeres (Evans and Noren Hooten, 2017). Numerous studies have demonstrated that inhibiting cell cycle can effectively prevent the proliferation and infiltration of malignant cells *via* cellular senescence. Moreover, this phenomenon has been associated with a range of medical ailments, including nonalcoholic steatohepatitis, diabetes mellitus, pulmonary arterial hypertension, osteoarthritis, and infectious diseases (Wei and Ji, 2018). Current research on senescence-associated genes (SAGs) focuses primarily on extending lifespan and improving health of patients (Ashraf et al., 2016; Xiang et al., 2019)A recent article hinted at the role of cellular senescence in sepsis, with RNA-seq analysis showing that multiple cellular senescence genes are involved in sepsis. In line with this, the expression of cellular senescence related genes p53 and p21 were upregulated (Chen et al., 2023). Metformin, acting as an anti-aging agent, alleviated cellular senescence in mouse myoblasts and skeletal muscle during sepsis as well as sepsis-associated liver injury and inflammatory response (Song et al., 2022). However, there is relatively little research on SAGs in spesis

This study explored the regulatory role of specific SAGs in the pathogenesis of sepsis and devised a publicly accessible diagnostic framework for sepsis using RNA-seq and scRNA-seq data. The current findings unveiled the interplay between the immune milieu of sepsis and SAGs, offering a novel direction for identifying effective diagnostic indicators for clinical interventions in sepsis.

## Method

### The expression of SAGs in peripheral blood between sepsis patients and a control group of healthy individuals

We downloaded two gene expression profiles of human peripheral blood (GSE28750, GSE57065) from the Gene Expression Omnibus (GEO) database. Batch effect was removed procedure using the “sva” tools in R software to create a produce database consisting of 92 sepsis samples and 45 healthy control samples. Latest SAGs identified by Saul et al. (2022) including senescence associated secretory phenotype (SASP) (*n* = 83), trans-membrane (*n* = 20) and intra-cellular (*n* = 22) proteins were used for further analysis. Subsequently, differential expression of these genes in sepsis and healthy samples was analyzed applying the “limma” package with the threshold value of abs (logFC) > 0.585 and adj.P.Val<0.05. Data were subsequently visualized employing the “ggplots” and “heatmap” packages.

### Discovering hub genes associated with senescence in sepsis

The relevance of each SAG was ranked by a support vector machine‐recursive feature elimination (SVM-RFE) method and “*randomForest*” with the R package “*randomForest*”. The top ten genes were selected and the central SAGs were identified by intersecting the top 10 SAGs determined by the two methods mentioned.

### Construction of a nomogram and receiver operator characteristic curve

Multivariate logistic regression analysis was conducted on the hub SAGs, based on which a nomogram for estimating the risk of developing sepsis was created. Furthermore, calibration curve and decision curve were plotted to assess the model stability. Internal validation of the model was performed using the bootstrap algorithm.

### Examining the relationship between the hub SAGs and infiltration of immune cells

The proportion of immune cells was assessed using “relative” and “absolute” methodologies in CIBERSORT. The aim of this section was to investigate the relationship between the hub genes and infiltration of 22 immune cell types. Additionally, the association between central genes and the quantities of 8immune cell categories in the peripheral blood derived from sepsis patients was analyzed using the software “MCP-counter".

### Consensus clustering analysis and co-expression analysis

The “*ConsensusClusterPlus*” algorithm was employed to determine the maximum number of cluster genes in human peripheral blood samples from sepsis patients, with a fixed value of 10. The top 5,000 genes showing the greatest variability were assessed, and the blood samples of sepsis were categorized based on their median absolute deviation. The WGCNA method was employed with standard setting to identify clusters of genes with similar mRNA expression patterns in sepsis patients.

### Gene ontology (GO) enrichment analysis

Co-expression clusters enriched with “Biological Process” terms of GO were assessed using the “fgsea” package.

### ScRNA-seq analysis

The scRNA-seq data (GSE167363) of healthy individuals and individuals with Gram-negative sepsis were obtained from the GEO database. Data analysis was conducted using the “Seurat” and “SingleR” packages. Scaled data were integrated using the “Harmony” function. Unbiased and impartial subsets of cells were obtained using the functions “FindNeighbors”, “FindClusters”, and “RunTSNE”. Cell types were characterized using the “SingleR” program. Subsequently, we employed the “AddModuleScore” function to calculate the signature-specific score for individual cells while considering the involvement of hub genes. The distribution of hub genes associated with aging across various cell types was analyzed.

Using the “Monocle2” package, we first isolated all NK cells and then selected those with an average expression greater than 0.1 and empirical dispersion surpassing 1 * dispersion fit. Cellular dimension was reduced using “DDRTree” tool, and the “reduceDimension” function was utilized to examine the cellular differentiation state. The visualization of NK cell differentiation trajectories was completed applying the “plot_cell_trajectory” function. Subsequently, NK cells were classified into high- and low-scoring groups according to the median score of senescence-associated hub genes as the basis for categorization. We used the “*CellChat*” R package to study the communication interaction between two groups of NK cells and other cells and to reveal the mechanism of communication molecules at single-cell level.

### Statistical analysis

Raw data were processed using R program (version 4.2.1). To identify the significant distinction between two groups, the Student’s t-test or Wilcoxon’s rank sum test was employed. The Kruskal–Wallis test was utilized to determine the dissimilarity among multiple groups. Statistically significant was defined as *p* < 0.05.

## Results

### Determination of differentially expressed SAGs in sepsis

**FIGURE 1:**
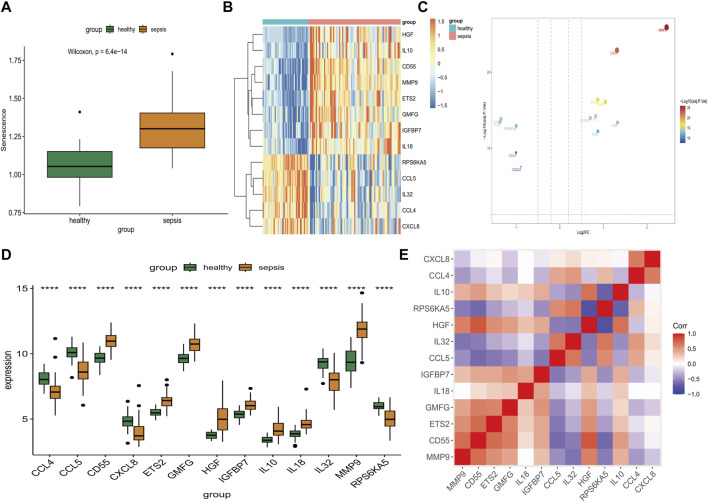
Identification of differential expression of SAGs. **(A)** The expression of SAGs in samples from healthy and septic patients. **(B)** A volcano plot showing the differential expression of SAGs between healthy and sepsis samples. **(C)** Differences in SAG expression between sepsis and healthy samples are displayed in this heat map. **(D)** The difference in expression of each SAG between healthy and sepsis samples can be seen in the histogram below. **(E)** Correlation of expression of SAGs in sepsis samples. *p < 0.05, **p < 0.01, ***p < 0.001 ****p < 0.0001.

After eliminating batch effect between GSE28750 and GSE57065, we successfully merged a database comprising 92 sepsis and 45 healthy control gene expression profile samples (Supplementary Figure S1). The expression level of SAGs in the sepsis samples exhibited a statistically significant increase (*p* = 6.4*10–14) (Figure 1A). Next, we used limma analysis to determine differentially expressed SAGs. As showed in Figure 1B, 8 specific SAGs including HGF, IL10, CD55, MMP9, ETS2, GMFG, IGFBP7, and IL18 were upregulated in the peripheral blood of sepsis, whereas 5 other SAGs (RPS6KA5, CCL5, IL32, CCL4, and CXCL8) were downregulated. The volcano map also showed 13 differentially expressed genes between the sepsis group and the healthy control group (Figure 1C). Variation in expression of SAGs between the two groups was depicted in Figure 1D. Additionally, Figure 1E presents the correlation among SAGs in sepsis samples. Moreover, MMP9 gene expression was positively correlated to CD55, ETS2 and GMFG expressions, respectively (Supplementary Figures S2A, B).

### Identification of eight senescence-associated hub genes in sepsis

**FIGURE 2:**
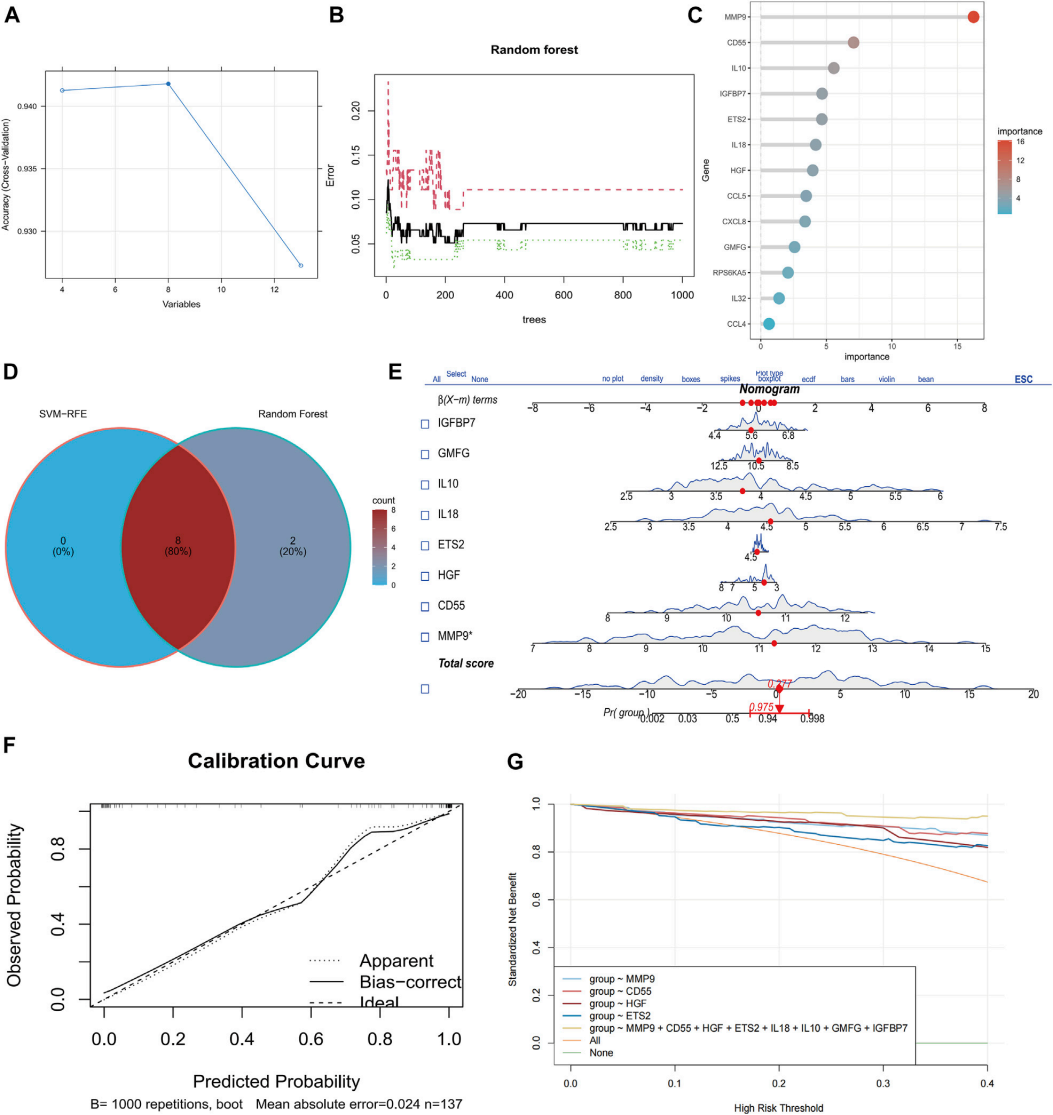
Identification of eight senescence-associated hub genes in sepsis. **(A)** SVM-RFE analysis of SAGs. **(B)** Random Forest analysis of SAGs. **(C)** The importance rank of SAGs shown in Bubble chart. **(D)** Hub genes were identified by intersection of random forest and SVM-RFE analysis. **(E)** Nomogram for the diagnosis of sepsis patients. **(F)** Calibration curve for the model. **(G)** Model evaluation curves.

To examine primary genes associated with senescence in sepsis, we employed the SVM-RFE algorithm and conducted Random Forest analysis to assess the significance of SAGs. The SVM-RFE algorithm was used to identify a subset of 8 features in the determined hub genes (Figure 2A). The relationship between error rate and number of taxonomic trees was used to reveal genes with relative importance greater than 2.5 as the key genes (10 genes) (Figure 2B). The important order of the out-of-bag scores for the 13 differentially expressed SAGs is displayed in Figure 2C. The marker genes obtained from SVM‐RFE models and Random Forest analysis were intersected, and 8 genes (IGFBP7, GMFG, IL10, IL18, ETS2, HGF, CD55, and MMP9) were identified for subsequent analysis (Figure 2D). These hub genes were employed to construct a nomogram (Figure 2E). The calibration data showed that the predicted probability of nomogram was closed to ideal curve (Figure 2F). DCA analyses demonstrated that the nomogram model had strong predictive ability (Figure 2G).

### The correlation of senescence-associated hub genes with and immune microenvironment (IME) in sepsis

**FIGURE 3:**
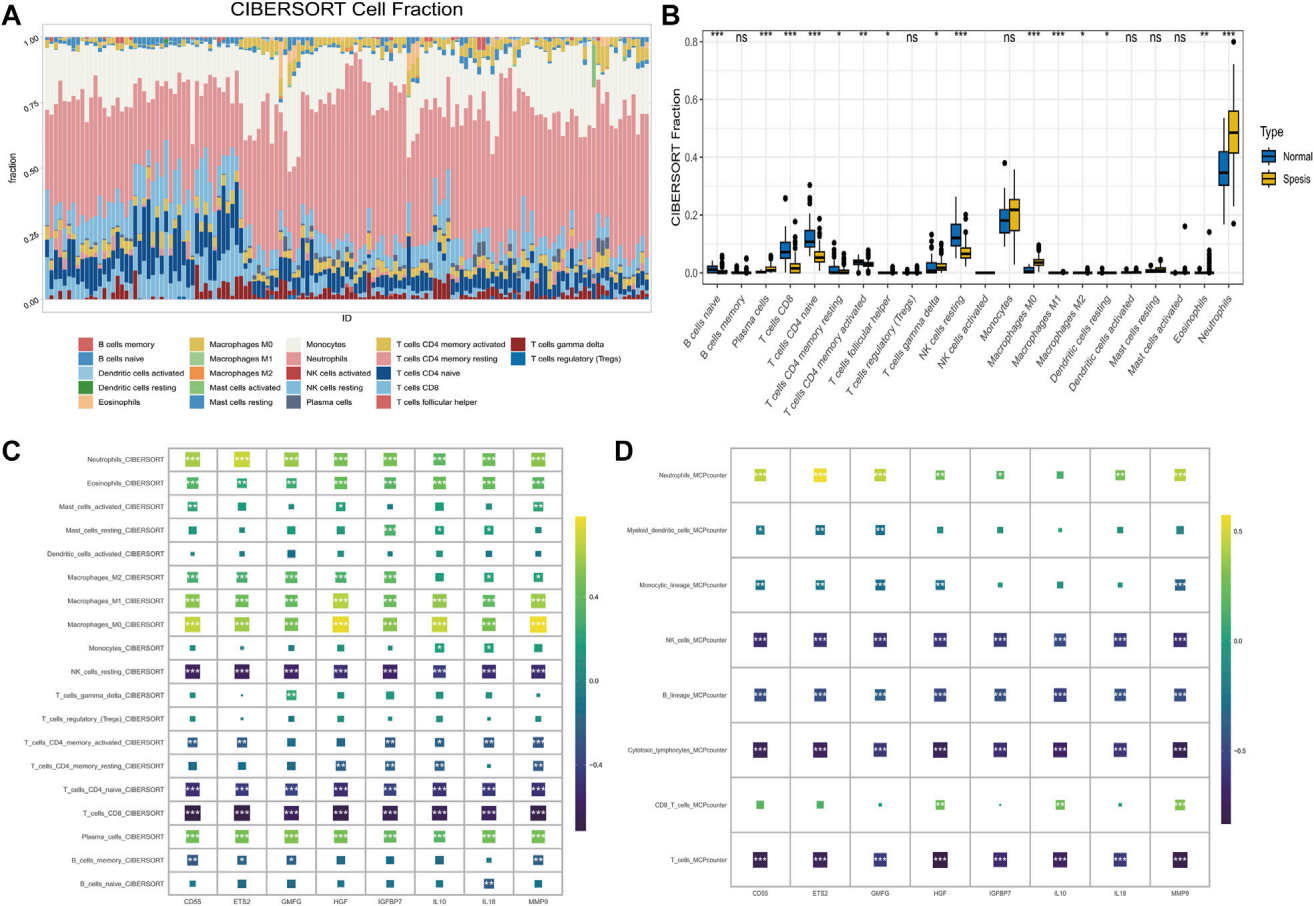
The correlation of senescence-associated hub genes with the IME in sepsis. **(A)** The percentage of immune cells in each sample. The ordinates represent percentages. **(B)** The proportions of immune cells in healthy and sepsis sample. **(C)** The correlation between 8 hub genes and 19 immune-related cells was analyzed using CIBERSORT **(D)** Correlation between hub genes and the abundance of 8 immune-related cells (MCP-counter).

Visualized immune cell distribution in both healthy and sepsis samples with color-coded bars is shown in Figure 3A. Notably, sepsis patients had significantly elevated proportions of plasma cells, macrophages M0, and neutrophils in comparison to the healthy group (Figure 3B). Through CIBERSORT analysis, NK cells, CD8+T cells were negatively correlated to 8 hub genes, while phagocytes, such as macrophages were positively associated with 8 hub genes (Figure 3C). Similarly phenomenon was observed in MCPcounter analysis (Figure 3D). MMP9 and ETS2 gene expressions were positively correlated to Neutrophils_MCPcounter (Supplementary Figures S2D, E).

### Comparison of age, state of SAPSII scores, gender, inflammatory and immune characteristics between two clusters

**FIGURE 4:**
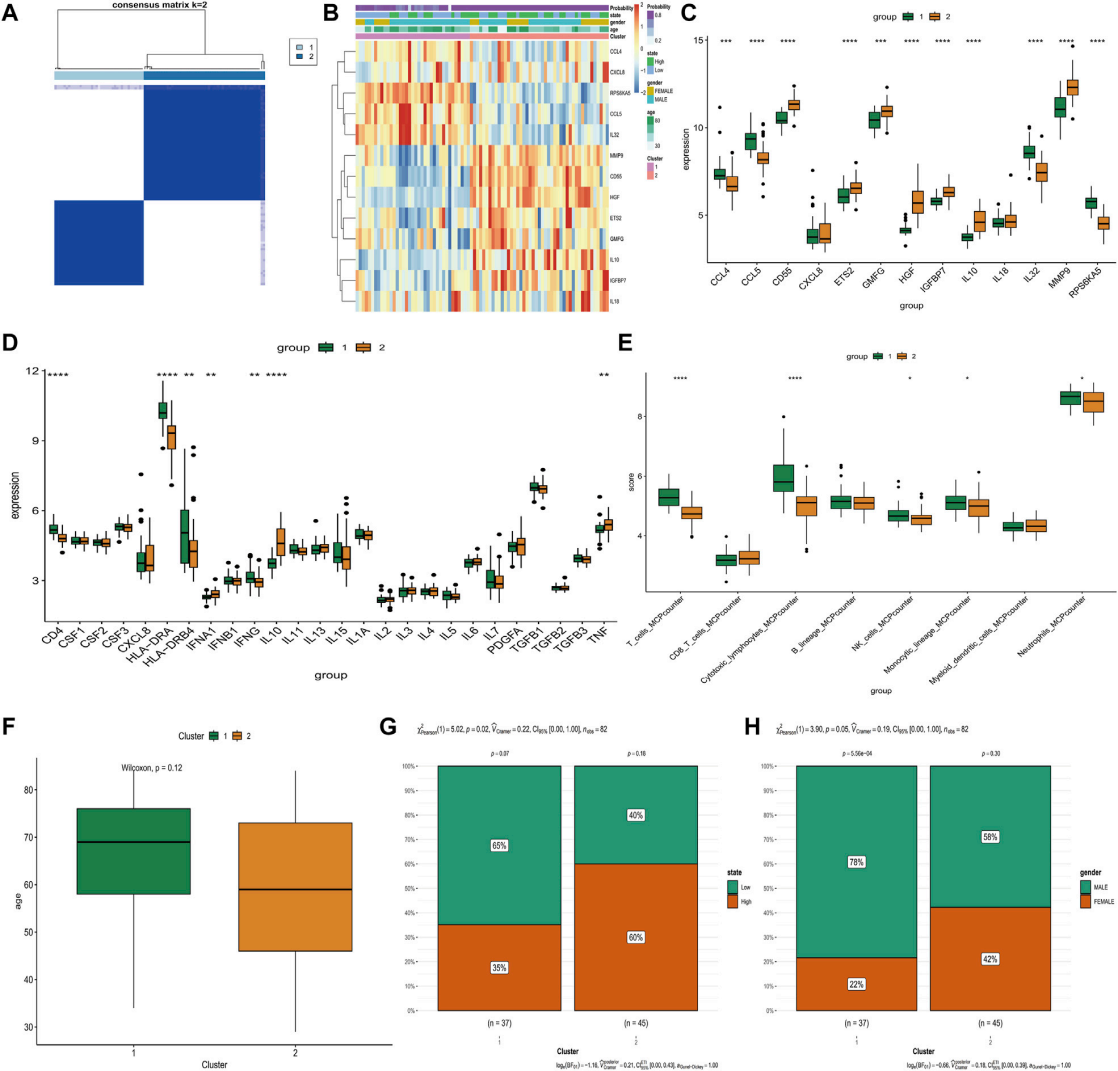
Comparison of various clinical and biologic characteristics between Two Clusters. **(A)** Consensus matrix plots depicting consensus values ordered by consensus clustering. **(B)** The distribution of distribution gene expression, state of SAPSII scores, gender and age in two clusters. **(C)** Differential expression of 13 differentially expressed SAGs in two clusters. **(D)** Differential expressions level of inflammatory cytokines in two clusters. **(E)** The abundance of 8 immune-related cells differs between the two clusters. **(F)** There were differences in age distribution between two clusters. **(G)** Cluster 2 had more samples with high SAPSII scores. **(H)** cluster 2 had more male samples. *p < 0.05, **p < 0.01, ***p < 0.001 ****p < 0.0001.

Consensus cluster analysis we successfully classified sepsis patients into two distinct groups based on the expression of 8 hub genes (IGFBP7, GMFG, IL10, IL18, ETS2, HGF, CD55, and MMP9) (Figure 4A). The expression of these hub genes varied significantly between the two clusters (Figure 4B). Specifically, CD55, ETS2, GMFG, HGF, IGFBP7, IL10, IL18, MMP9, and TNF were upregulated, while CCL4, CCL5, IL32, RPS6KA5, CD4, HLA-DRA, HLA-DRB4, IFNG, T cells, cytotoxic lymphocytes, NK cells, monocytic lineage, and neutrophils were downregulated in cluster 2 (Figures 4C–E). In addition, cluster 2 contained younger sepsis patients than cluster 1 (Figure 4F). The proportion of sepsis patients with higher SAPSII scores was significantly more in cluster 2 than in cluster 1 (Figure 4G), but gender did not differ between the two clusters (Figure 4H). The clinical information of database was showed in Supplementary Table S1.

### Establishment of a co-expression network

**FIGURE 5:**
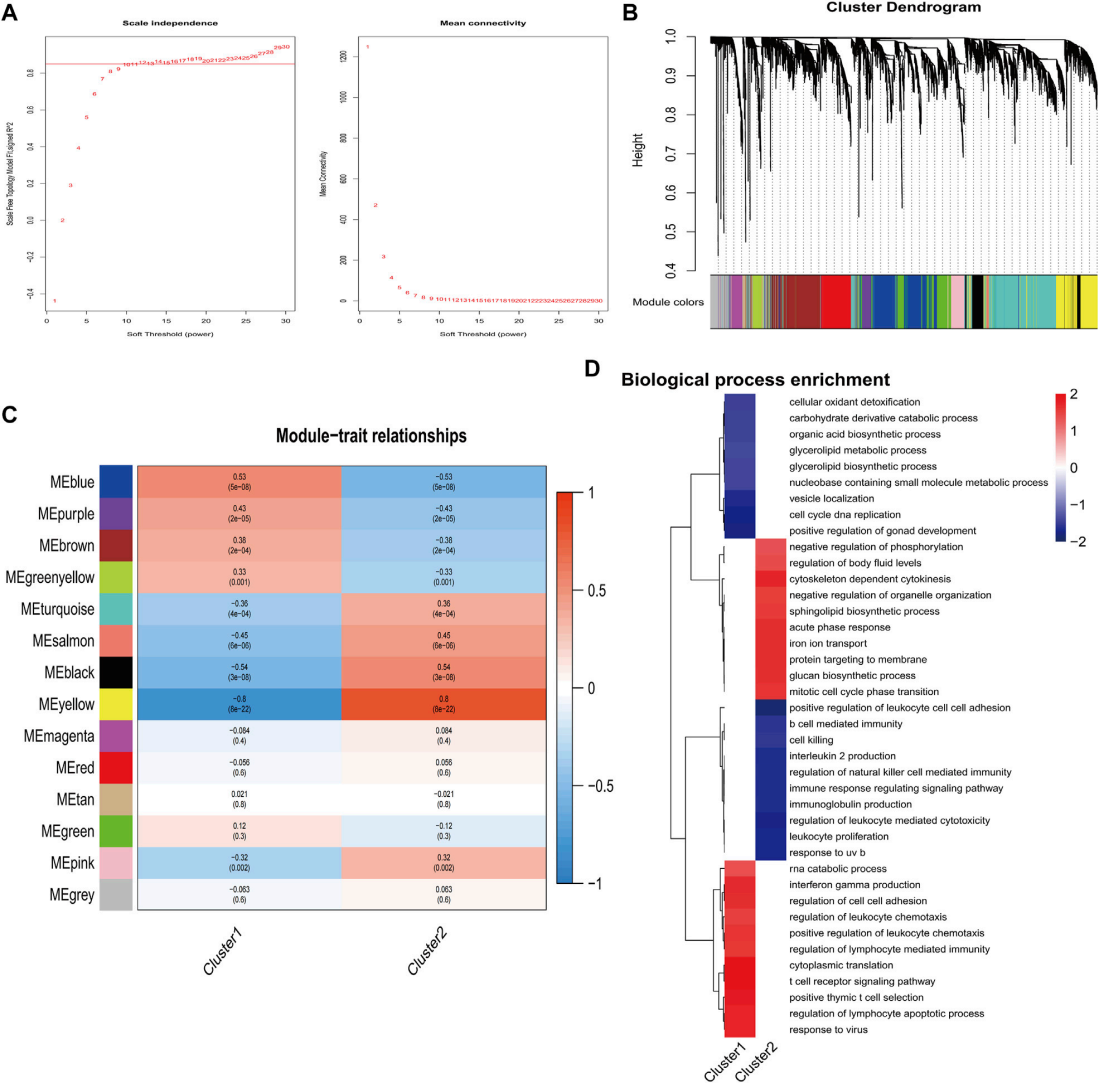
Identification of important modules correlated with hub genes. **(A)** Calculation and determination of the optimal soft-threshold power. **(B)** Genes’ clustering dendrogram based on topological overlap. **(C)** The correlation between module eigengenes and two clusters. **(D)** Analysis of functional enrichment for two clusters.

By performing dynamic tree-cut analysis, co-expression modules were identified with a soft threshold of 10 serving as the most suitable SFT for the construction of a scale-free network (Figure 5A). Subsequently, dynamic module identification was performed in combination dataset, with the number of genes per module not less than 50 (Figure 5B). Interestingly, cluster 1 and cluster 2 had the exact opposite properties with modules (Figure 5C). Next, we performed GO enrichment analysis on clusters. Cluster 1 exhibited a positive correlation with the regulation of leukocyte chemotaxis, while cluster 2 demonstrated a negative correlation with the regulation of natural killer cell-mediated immunity (Figure 5D).

### Single-cell analysis revealed the interaction between the SAGs and immune in sepsis

**FIGURE 6:**
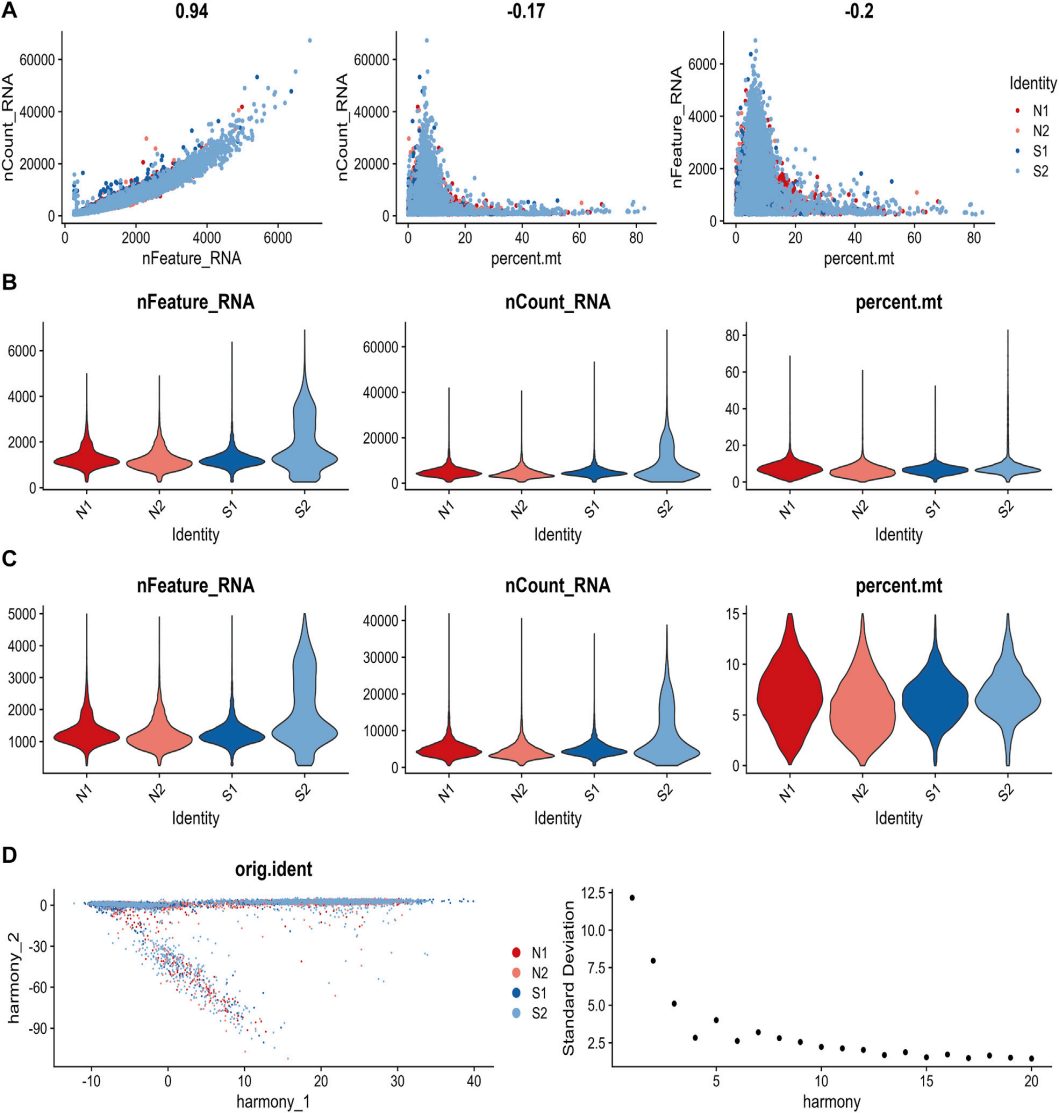
The process of quality control for single-cell data. **(A)** The relationship between gene expression, cell counts, and mitochondrial content were examined in each sample. **(B)** The values of nCount RNA, nFeature RNA, and percent. mt for each sample before filtering were displayed. **(C)** The values of nCount RNA, nFeature RNA, and percent. mt for each sample after filtering was showed. **(D)** Principal Component Analysis (PCA) plot and an elbow plot were presented.

After pre-processing scRNA-seq data from GSE167363 based on the stringent quality control metrics described, 26,191 high-quality cell samples were separated. The correlation coefficient between nCount RNA and nFeature RNA, which serves as a measure of unique molecular identifiers, was 0.94, as shown in Figure 6A. The number of genes detected (nFeature) and the depth of sequencing (total UMI, nCount), and the percentage of mitochondrial genes (percent.mt) were plotted before and after cell filtration (Figures 6B, C). Additionally, principal component analysis (PCA) was conducted to selected the top 20 principal components for subsequent analysis (Figure 6D).

**FIGURE 7:**
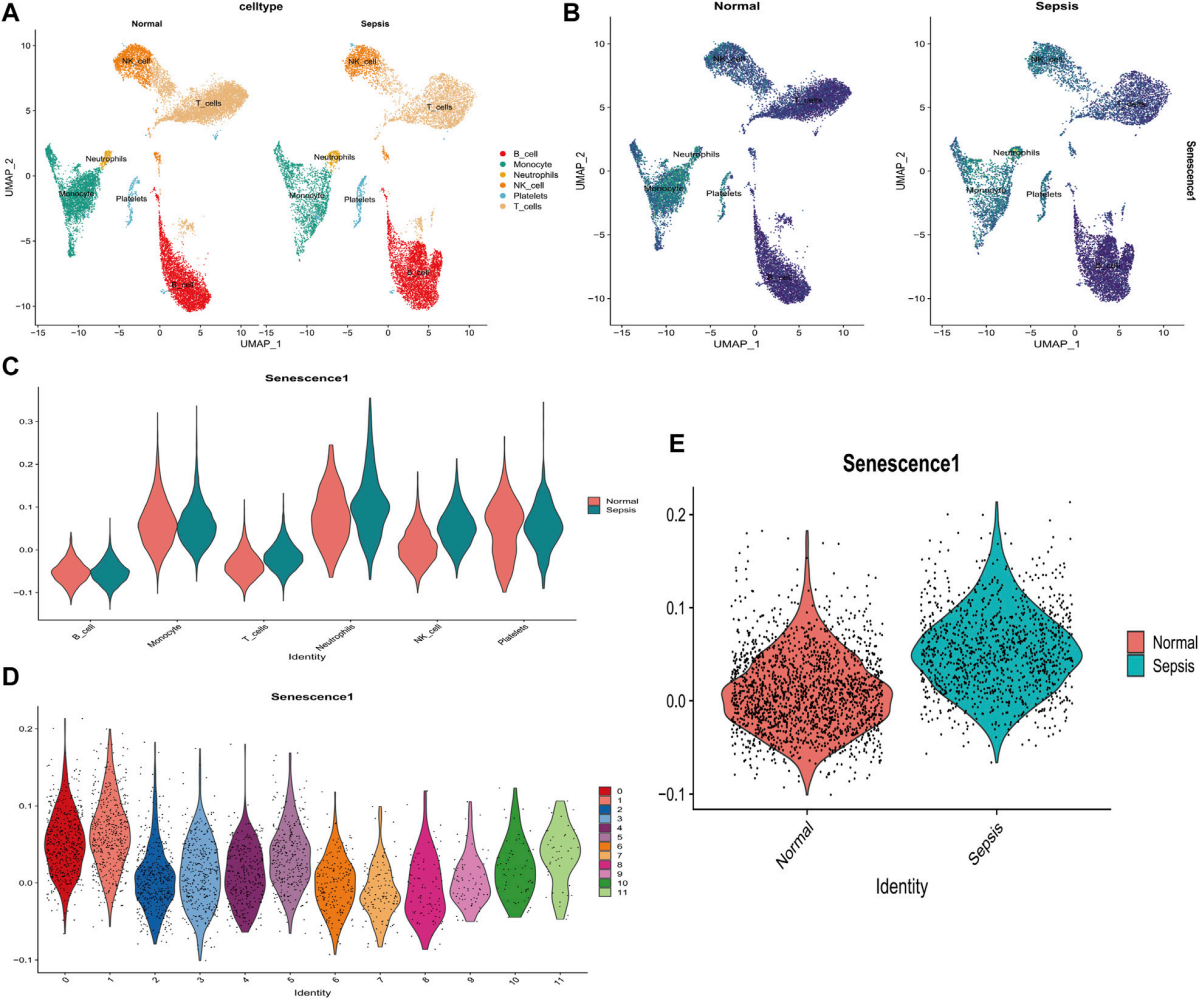
Analysis of the relationship between SAGs and the immune system in sepsis using single cells. **(A)** UMAP displays cell subpopulations in patients with and without sepsis. **(B)** The distribution of numerous hub genes associated with senescence across various cell types. **(C)** The violin diagram illustrates the differential expression of a large number of hub genes associated with senescence in various immune cells from healthy and sepsis samples. **(D)** The violin diagram depicts the distribution of senescence-associated hub gene scores among various subtypes of NK cells in sepsis samples. **(E)** Levels of senescence-associated hub genes differ between healthy and sepsis patients’ NK cells. *p < 0.05, **p < 0.01, ***p < 0.001 ****p < 0.0001.

Single R annotation identified six distinct cell subtypes between healthy and sepsis samples, namely, B cells, monocytes, neutrophils, NK cells, platelets, and T-cells, were (Figures 7A, B). Notably, the scores of senescence-associated hub genes in T cells (*p* < 2.2*10-16), neutrophils (*p* < 6.2*10-4), and NK cells (*p* < 2.2*10-16) of sepsis patients were markedly elevated compared to the healthy control group (Figure 7C). To more comprehensively examine the distribution of scores for senescence-associated hub genes in NK cells, this study subdivided NK cells into 12 distinct subclusters and subsequently analyzed the distribution of these scores within each subcluster (Figure 7D). Moreover, scores of senescence-associated hub genes in NK in sepsis samples was higher than that in normal samples (Figure 7E). Those data indicated that senescence-associated hub genes in immune cells may be closely related to the development of sepsis.

### Pseudo-time and cell-chat analysis of NK cells

**FIGURE 8:**
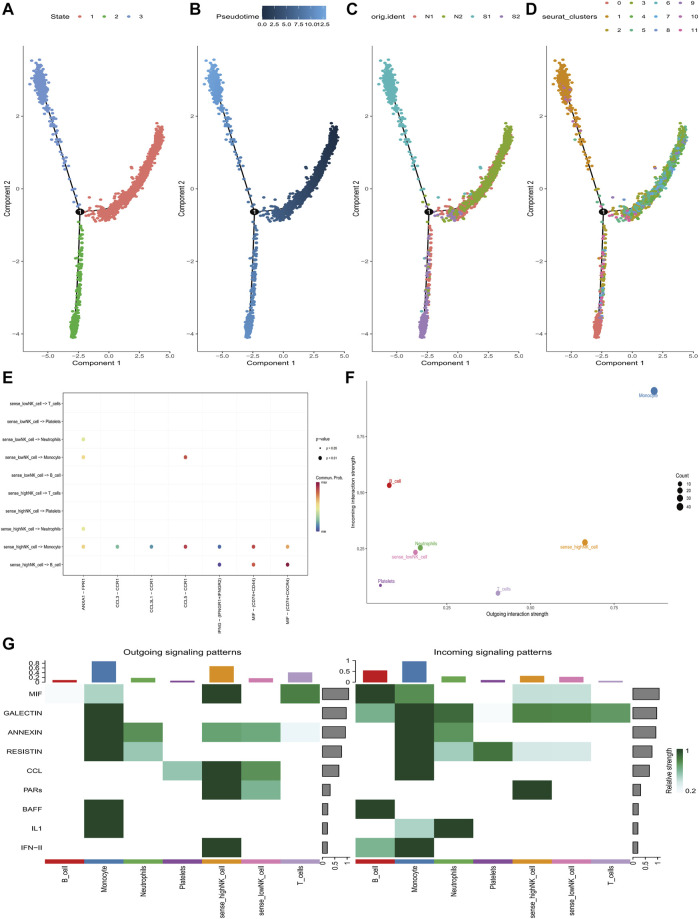
Pseudo-time and cell-chat analysis of NK cells in GSE167363. **(A)** Three differentiation phases of NK cells. State 1 is the initial differentiation phase. **(B)** Differential maturation times of NK cells. Darker blue represents an earlier stage of differentiation, whereas a paler blue represents a later stage of differentiation. **(C)** NK cell differentiation in four samples. **(D)** Differentiation of different NK cell subclusters. **(E)** The ligand-receptors that mediate communication between NK cells at different state of senescence-associated hub genes’ scores and different cell sub-populations. **(F)** The communication intensity of NK cells with different senescence-associated hub genes’ scores and other cell sub-populations. **(G)** Identification of global communication patterns and major signals for specific cell sub-populations.

Pseudo-temporal analysis was performed separately for NK cells annotated to explore their differentiation directions using the Monocle 2 algorithm. The results showed that NK cells gradually followed 3 differentiation directions (Figure 8A). Subsequently, simulations were conducted to trace the differentiation trajectories of all NK cells, and we observed that the intensity of blue coloration was inversely correlated with temporal differentiation, indicating a progressive differentiation of NK cells from right to left. Notably, the darkest blue corresponded to the earliest differentiated cells (Figure 8B). Furthermore, distinct differentiation trajectories for various samples and subclusters of NK cells were observed (Figures 8C, D). It was found that NK cells exhibiting varies scores of senescence-associated hub genes were regulated by distinct ligand receptor pathways during their interactions with other cells. When NK cells of high scores communicated with monocytes, IFNG- (IFNGR1+IFNGR2) was significantly downregulated and CCL5-CCR1, MIF- (CD74^+^CD44) were significantly upregulated. When it communicated with B cells, IFNG- (IFNGR1+IFNGR2) was significantly downregulated and MIF- (CD74^+^CD44), MIF- (CD74+CXCR4) were significantly upregulated (Figure 8E). NK cells with higher scores had strong signal output ability during the communication of various types of cells (Figures 8F, G).

## Discussion

Previous research has demonstrated a correlation between senescence and the onset of sepsis. Margotti et al. (2020) proposed that aging increases permeability of post-sepsis cerebral barrier, which may further promote infiltration of neutrophils in the central nervous system, leading to an increase in oxidative stress. Agudelo-Ochoa et al. (2020) found that loss of gut microbial diversity and changes in microbiota function due to senescence results in a higher risk of sepsis. Increasing formation of platelet-monocytes in geriatric patients diagnosed with sepsis is correlated with a greater death risk within a 28-day period (Rondina et al., 2014). However, the association of senescence-associated genes with sepsis has rarely been reported. In this study, we identified 8 senescence-related genes (IGFBP7, GMFG, IL10, IL18, ETS2, HGF, CD55, and MMP9) and developed a diagnostic model for the prediction of sepsis occurrence. These genes were associated with the immune microenvironment in sepsis.

In recent years, anti-inflammatory cytokines (Liu et al., 2022) and changes in cell surface markers in monocytes and lymphocytes (Faix, 2013) have been considered as markers for immunosuppressive phase of sepsis. Discovery of these biomarkers facilitates the diagnosis and early intervention of sepsis, while reducing the risk of death from the disease (Barichello et al., 2022). We identified 8 genes associated with senescence and used them to predict the onset of sepsis. Studies have shown that IGFBP7 is important for clinical staging of sepsis-related acute kidney injury (Molinari et al., 2022) because IGFBP7 can delay the progression of acute kidney injury by inhibiting RNF4/PARP1-mediated tubular injury (Yu et al., 2022). Changes in the concentration of [TIMP-2] [IGFBP7] in urine after fluid resuscitation could be used to differentiate the risk of developing sepsis for patients with acute kidney injury (Fiorentino et al., 2020). GMFG is currently studied in the field of oncology. RNF144A exerts a negative regulatory effect on the expression of GMFG in breast cancer by specifically targeting YY1-degrading proteasomes, thereby preventing the proliferation, migration, and invasion of breast cancer cells (Zhang et al., 2022). Similarly, in solid tumors such as colorectal cancer and ovarian cancer, GMFG promotes the development of cancer (Zuo et al., 2014; Wang et al., 2017). As a potent endogenous immunosuppressive cytokine (Scumpia and Moldawer, 2005), IL10 is considered as the most important factor associated with rapid death in patients with sepsis (Chuang et al., 2014). Gu et al. (2008) proved that the presence of polymorphism in the promoter region of IL-10 gene significantly influences the susceptibility to sepsis and subsequent outcome of sepsis. Moreover, IL-10 can stimulate immunosuppressive pathway of sepsis by promoting nuclear localization of S100A9 and MDSC development (Bah et al., 2018). Pro-inflammatory cytokine IL-18 has substantial potential in distinguishing Gram-negative from positive sepsis and may serve as an important marker for monitoring changes in sepsis (Tschoeke et al., 2006). Michelle V. Eidt et al. demonstrated that IL-18 has an accuracy in predicting fatal outcomes up to 88.9% and 90% in the severe sepsis and septic shock groups, respectively, with a sensitivity of 80% and 83% and a specificity of 100% (Eidt et al., 2016). ETS2 has been recognized as an effective prognostic biomarker for patients suffering from acute-on-chronic liver failure, and it mitigates liver failure through the downregulation of the HMGB1/lipopolysaccharide-induced inflammatory response (He et al., 2023). A high level of HGF is firmly associated with poor outcomes in sepsis patients (Peng et al., 2021). Elias et al. (2023) discovered that endothelial dysfunction resulted from sepsis stimulates the production of HGF, which activates C/EBP in liver cells and ultimately causes liver failure or acute-on-chronic liver failure. Upregulated C5a expression in the body exacerbates the progression of sepsis. CD55 expression on neutrophils can be inhibited through NOD2 or IL-10-mediated mechanism (Zamboni et al., 2013; Kim et al., 2014). Previous research showed that the upregulation of antagonistic MMP9 may serve as a viable therapeutic approach for individuals suffering from severe sepsis or septic shock (Sachwani et al., 2016). This is consistent with the result that MMP9 expression is associated with septic shock and is significantly correlated with sequential organ failure assessment scores in another study (Hong et al., 2022). In conclusion, the role of these 8 senescence-related genes in sepsis and other severe diseases provides an adequate theoretical foundation for our prediction model.

Currently, it is widely accepted that sepsis consists of two distinct phases, an initial phase characterized by immune activation and a subsequent phase characterized by chronic immunosuppression, which ultimately leads to cell death (Nedeva et al., 2019). During the early stages of pathogen detection, primary immune cells such as macrophages, dendritic cells, and neutrophils undergo rapid proliferation. Subsequently, adaptive immune system initiates the activation of T helper cells and cytotoxic T cells through T cell receptor activation. Subsequently, differentiation and proliferation of these cells contribute to the development of a highly specific adaptive immune response (Fitzgerald and Kagan, 2020). In the context of a severe infection, acute inflammatory response may result in immunosuppression if the immune system fails to defense effectively. Such immunosuppression is characterized by phenotypic alterations in systemic immune cells, which directly influences the generation of innate and adaptive immune cells and the functionality and viability of effector cells (Skrupky et al., 2011). Hence, activation of the immune system and induction of inflammation play a critical role in preventing infection in sepsis, necessitating a comprehensive comprehension and development of therapeutic interventions to enhance immune system homeostasis during sepsis treatment (Nedeva, 2021). In this study, single-cell sequencing analysis was performed on sepsis patients, and we discovered that senescence-related genes were significantly high-expressed in the NK cells of sepsis patients, and that NK cells with high-expressed senescence genes played a crucial role in the communication of immune cells. During sepsis, NK cells may be overactivated, leading to amplified systemic inflammation. Overproduction of IFN- and TNF as a result of overactivation causes excessive inflammation, multi-organ failure, and increased mortality (Guo et al., 2018). A majority of studies indicated that the number of NK cells in older adults remains relatively stable or slightly higher (Almeida-Oliveira et al., 2011; Campos et al., 2014), and the slight increase is caused by an increased proportion of more mature CD56^dim^ subpopulation, whereas the number of embryonic CD56^bright^ subsets decreases in older adults (Chidrawar et al., 2006). Age-related increase in the release of IFN- by NK cells stimulates tissue injury, which explains a higher prevalence of sepsis in older individuals (Xie et al., 2021). This is also consistent in our findings.

The current research also has limitations. The influencing factors of sepsis were primarily predicted at the molecular level, but relevant clinical variables were not included. Although this study demonstrated that SAGs may serve as diagnostic markers for sepsis and they interact with IME in sepsis, additional animal and clinical research is required to confirm our findings.

## Conclusion

A comprehensive analysis was conducted to investigate the impact of senescence-related genes on sepsis, and 8 hub genes (IGFBP7, GMFG, IL10, IL18, ETS2, HGF, CD55, MMP9) associated with senescence were identified. These genes manifested a strong potential to serve as diagnostic markers for patients suffering from sepsis. Notably, significant associations were observed between these genes with immune cells and inflammatory factors. This study proved novel insights into the interaction between sepsis and aging, potentially guiding future clinical treatment and diagnosis of sepsis.

## Data Availability

The original contributions presented in the study are included in the article/Supplementary Material, further inquiries can be directed to the corresponding author.

## Supplementary material

SUPPLEMENTARY FIGURE S1 Dataset quality control. **(A, B)** The integrated datasets were subjected to Principal Component Analysis (PCA) after de-batching **(C, D)** Before and after clustering of WGCNA analysis.

SUPPLEMENTARY FIGURE S2
**(A–C)**: The positive correlation between MMP9 with CD55, ETS2, GMFG, respectively. **(D, E)**: The positive correlation between Neutrophils_MCPcounter with MMP9, ETS2, respectively.

SUPPLEMENTARY TABLE S1 The clinical information of database.
