# Supplementary figures and images for: Comprehensive analysis of senescence-associated genes in sepsis based on bulk and single-cell sequencing data
Source: Front Mol Biosci. 2024 Jan 8;10:1322221. doi: 10.3389/fmolb.2023.1322221 (PMC10801732; doi:10.3389/fmolb.2023.1322221)

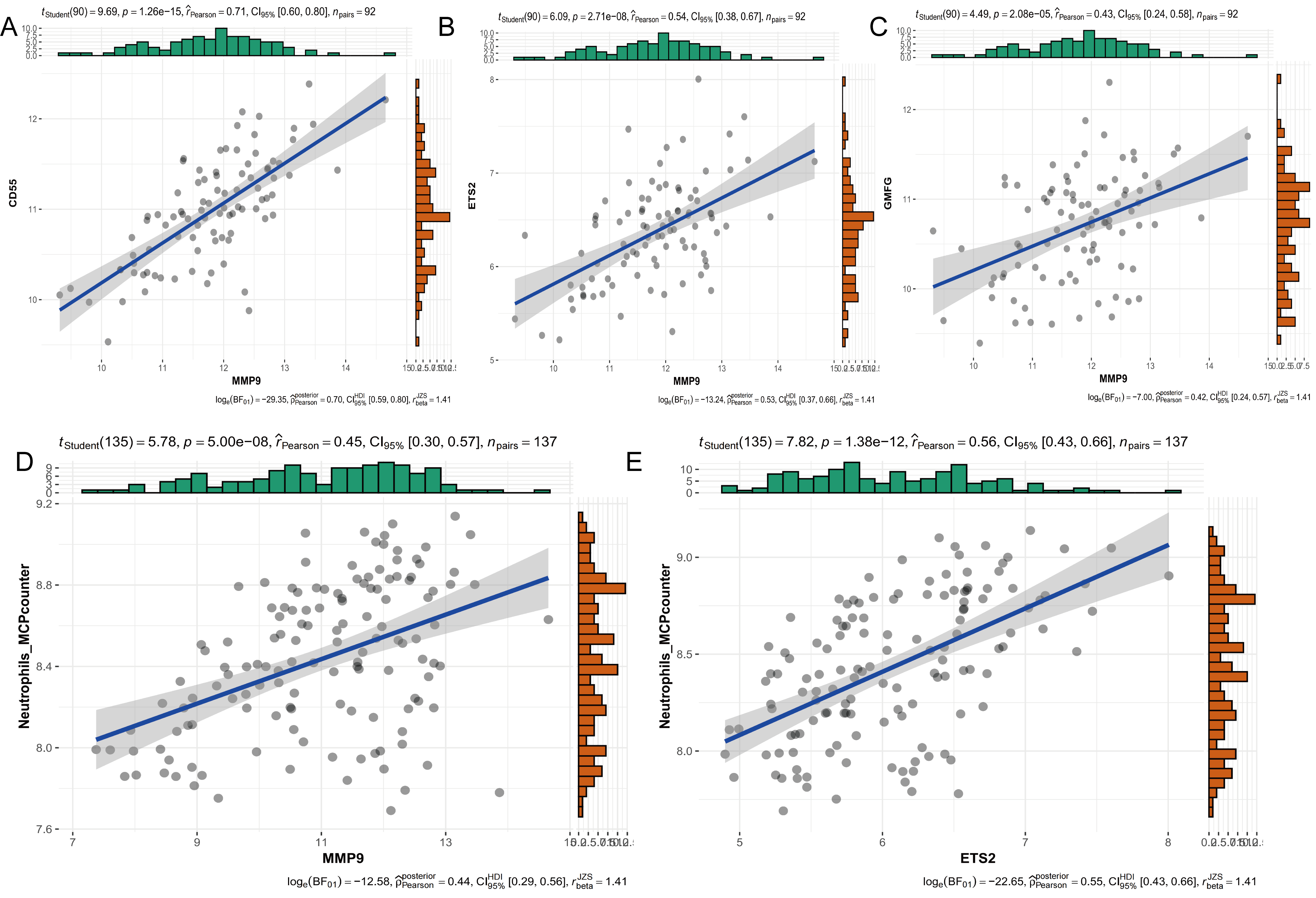

Supplement: Supplementary file 1 [file Image2.TIF]

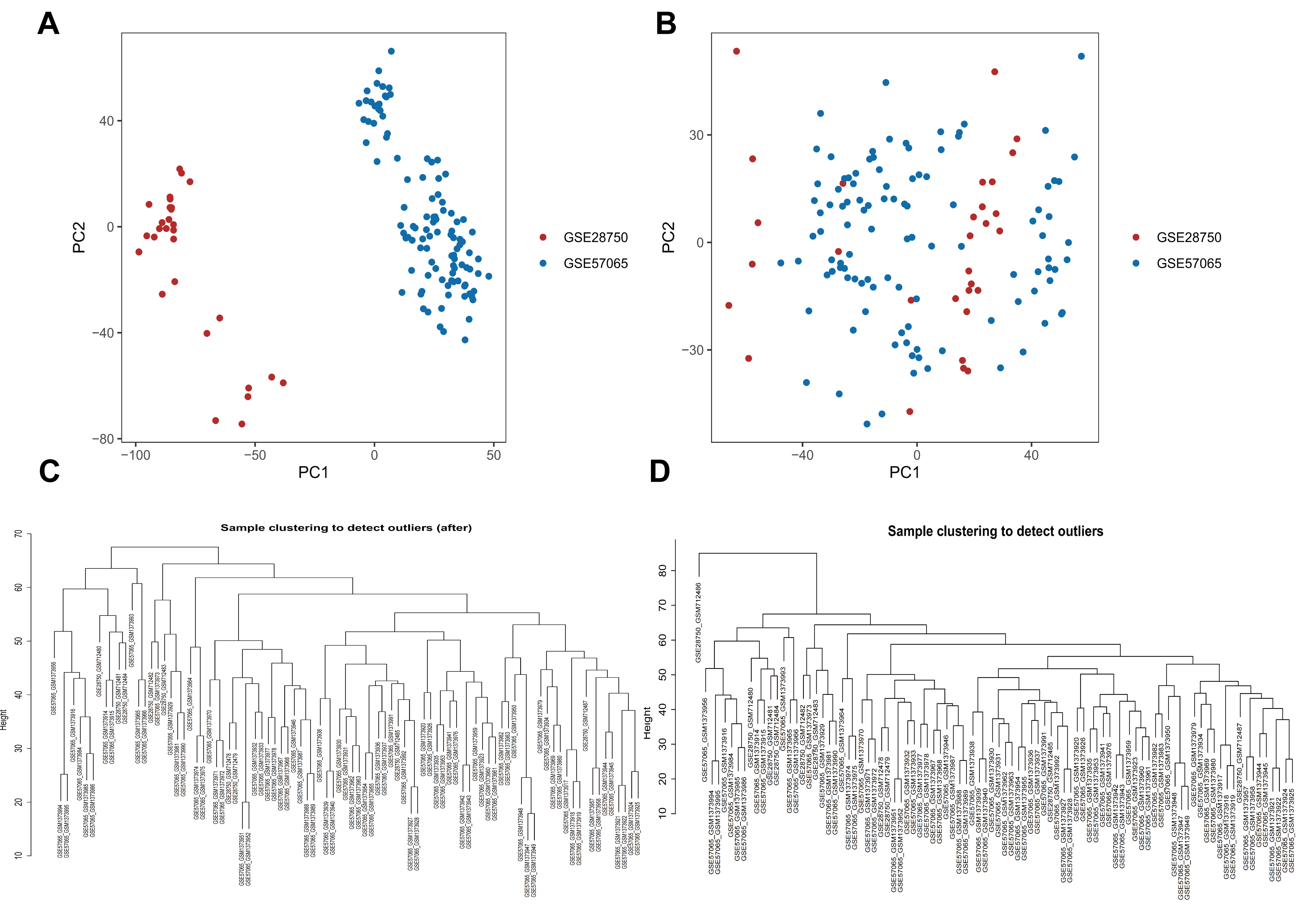

Supplement: Supplementary file 2 [file Image1.TIF]
